# Supplementary material for: 4-aminopyridine improves evoked potentials and ambulation in the taiep rat: A model of hypomyelination with atrophy of basal ganglia and cerebellum
Source: PLoS One. 2024 Mar 1;19(3):e0298208. doi: 10.1371/journal.pone.0298208 (PMC10906851; doi:10.1371/journal.pone.0298208)
Supplement: Attachment — Submitted filename: REVIEWERS ANSWERS.docx [file pone.0298208.s001.docx]

**Reviewer 1.**

1. However, as the first sentence of the abstract indicates, the taiep rats have hypomyelination. And hypomyelination reduces the conduction velocity of action potentials as expected. Therefore, it is not clear what is the novel point of this study?.

**REPLY:** We previously demonstrated a progressive deterioration of conduction velocity in the optic nerve under *in vitro* conditions (Roncagliolo et al., 2006), and this reduction correlated with the reduction of myelin thickness (Lunn et al., 1997). The present study is the first study *in vivo* and we add the analysis of the effects of 4-aminopyridine (4-AP) in *taiep* rats a model of hypomyelination with atrophy of the basal ganglia and cerebellum (H-ABC). Finally, we compare our results of the somatosensory evoked potentials with the first reported Mexican patient with H-ABC.

Our results could be the first step to test 4-AP in human patients with H-ABC or even with others leukodystrophies or leukoencephalopathies as a therapeutic agent.

2. In addition, the manuscript, especially the result parts need to be better written. Authors do not explain what their observation indicates. They describe the results but do not interpret these data in deep. Authors may give a short summary or conclusion of each results, and describe what do these changes indicate or suggest. I strongly recommend the authors go through the text carefully and thoroughly to write it in a more professional manner.

**REPLY:** We really appreciate the observation did by the reviewer. We rewritten several paragraphs, and all the Ms was reviewed by a native English speaker. We did all experiments rigorously and the corresponding analysis following the best practices in scientific research. But, in base of your suggestion, we review all the Ms carefully in order to improve its quality.

3. **REPLY:** Thank you for the observation. Now in each section of the Results section we added a short summary to emphasize the experimental data obtained.
4. In addition, in figure 1, both wt and mutant mice showed similar amplitude, but in figure 3, the amplitude of controls are almonst four times higher than that of the mutant mice without 4-AP treatment. Could the authors explain the discrepancy between the control data in these two figures?

**REPLY:** We really appreciate your observation. The results obtained are in rats not in mice. We change Figure 1 to see the distribution of the somatosensory evoked potentials along lumbar and the lowest thoracic spinal cord levels. All evoked potentials had similar amplitudes, only we changed the scale to emphasize our results.

5. Authors may add tags to indicate N2 in figure 1 or give a scheme of N1 and N2 in figure 2.

**REPLY:** The observation is quite useful and now we added the suggested tags in the N1 and N2 waves of the somatosensory evoked potentials (SSEPs) on Figure 1 and in Figure 2 as suggested.

We also change the description in the results section and the corresponding Figure legends of both figures.

6. In line 452 and 456, fluorescence intensity of what? Myelin or NF?

**REPLY:** It is a very useful observation, we appreciate it. Now fluorescence intensity refers to myelin. We rewritten the paragraph to be clearer and it is also the case in Fig. 8 and the corresponding figure legend.

**Reviewer 2.**

I must commend your work on this well-written article which is a great addition to the body of knowledge. The article gives deep insight that will most likely come in handy in the diagnosis and management of patients with H-ABC. I particularly like that your methods section was detailed and very clear in a manner that can be replicable. Well done once again.

**REPLY:**  We really appreciate his/her comments. We feel motivated to be extremely careful with the methods in future manuscripts.

**Reviewer 3.**

1. lines 80 and 81: despite it may be true to use fore and hind limb, considering a human standing in a standard anatomical posture, it's good to use lower and upper extremities instead of forelimb and hindlimb.

**REPLY:** We follow up the suggestion and now we refer to lower and upper extremities along the manuscript.

2. Please recheck all keywords considering the mesh.

**REPLY:** Thank you for the observation. We change the keyworks to be more assertive and we will have more impact in the scientific community.

3. PLEASE make a revision about grammatical structures and dictation, for example at the last paragraph of introduction, lines 154 and 155 "the aim of this study we analyze..." there is no agreement between noun and the verb. And in line 450 there is dictation error.

**REPLY:** This is very good observation and I really appreciated it. Now we rewritten the aim of the study and in line 450 we also rewrite the paragraph. We review carefully all the Ms and a native English speaker editing the English language text.

4. lines 181-184-352: the reference using which you have adjusted the dose of anesthetic drug must be mentioned.

**REPLY:** We added references to adequate level of anesthesia in Material and Method section through all experimental session. I added the paper of Nery, 1968.

5. line 185: the size of catheter (thickness and outer diameter) must be mentioned, for instance gauge 22.

**REPLY:** Thank you for your observation we added the size of the catheter use for injection of fluids 22 gauge (0.71 mm).

6. lines 187 and 215: information about product such as manufacturer company should be said.

**REPLY:** The silver electrode was purchased from (WPI, Inc., USA) and we added in both paragraphs as suggested.

7. lines 191 and 192: it's crucial to name or cite the source of information using which you have distinguished the exact region. for example, Paxinos atlas or other references**.**

**REPLY:** Thank you for the observation we added a phrase indicating that we follow up the stereotaxic coordinates of the stereotaxic atlas of Paxinos and Watson (2013).

8. lines 234 and 235: the model number of the camera should be addressed.

**REPLY:** We used a Nikon stereomicroscope model_______ and we draw the area of cortical stimulation in an image of the stereotaxic lamina using in the antero-posterior and medio-lateral coordinates.

9. lines 239-246: The entire paragraph related to the behavioral testing process lacks reference and should be revised. additionally, the measure of room light that can affect rat behavioral factors must have been measured and reported in lux.

**REPLY:** Thank you for your observation. We added a reference related to novelty-induced ambulation test by Padilla et al., 2010. The intensity of light during this procedure was 149 lux and it was added to the corresponding paragraph.

10. lines 250-255: as before, in the paragraph pertaining to Drugs, there was no citation about the references that based on them the drug adjustment was made.

**REPLY:** We already had the reference 41 from Peña and Tapia 1999 who made *in vivo* rats using microdyalisis in the hippocampus during different 4-AP doses. In base of that we used the 1 mg/Kg dose in order to obtain adequate results avoiding but avoiding epilepsy crisis.

11. line 268: this section of the article (Tissue preparation and image acquisition) should go under revision because of the lack of citations about protocol.

**REPLY:** We added references of the protocol in base of Alata et al., 2021.

12. line 271: The dosage of the anesthetic used in the anesthesia process before the preparation of the tissue was wrongly mentioned, and usually the dosage of ketamine should be about ten times that of xylazine.

**REPLY:** Thank you for your observation. It was a mistake now the doses of ketamine and xylazine were adjusted.

13. At the section" Morphological characteristics of the spinal cord" What protocol and software is used to quantify data related to immunostaining images? Software similar to ImageJ and the like?

**REPLY:** Thank you for your observation. Now we added “The protocol used for tissue preparation was described previously (Alata et al., 2021). Briefly three *taiep* and three WT rats aged three months were anesthetized with a mixture of ketamine/xylazine…”. We used FIJI software to analyze and measure myelin immunostaining. In base on ImageJ but this software offers additional capabilities. We also added the reference Schindelin, 2012.

14. There are many outdated references In the Reference section, and are from the 1980s. Please replace with more up-to-date sources.

**REPLY:** Thank you for your observation. We did a detail review of the literature. However, there are only a few studies using clinical neurophysiology such as evoked potentials. Probably because now the clinicians preferred MRI and other imageology techniques.
